# Monitored and Modeled Ambient Air Concentrations of Ethylene Oxide: Contextualizing Health Risk for Potentially Exposed Populations in Georgia

**DOI:** 10.3390/ijerph19063364

**Published:** 2022-03-12

**Authors:** Ryan C. Lewis, Patrick J. Sheehan, Christopher G. DesAutels, Heather N. Watson, Christopher R. Kirman

**Affiliations:** 1 Health Sciences, Exponent, Inc., Oakland, CA 94612, USA; psheehan@exponent.com; 2 Health Sciences, Exponent, Inc., Maynard, MA 01754, USA; cdesautels@exponent.com; 3 Data Sciences, Exponent, Inc., Menlo Park, CA 94025, USA; hwatson@exponent.com; 4 Summit Toxicology, Bozeman, MT 59722, USA; ckirman@summittoxicology.com

**Keywords:** ethylene oxide, monitoring, modeling, exposure metrics, endogenous equivalent concentration, total equivalent concentration, contextualization, exposure science

## Abstract

Recent studies have monitored and modeled long-term ambient air concentrations of ethylene oxide (EO) around emitting facilities in Georgia with the intent of informing risk management of potentially exposed nearby residential populations. Providing health context for these data is challenging because the U.S. Environmental Protection Agency’s risk-specific concentrations lack practical utility in distinguishing a health significant increase in exposure. This study analyzes EO data for eight emitting facilities, using a previously published alternative exposure metric, the total equivalent concentration, which is based on U.S. Centers for Disease Control biomarker data for the non-smoking U.S. population. Mean concentrations for monitoring sites were compared to mean background concentrations to assess whether emissions contribute significantly to environmental concentrations. To assess the health significance of potential exposure at nearby residential locations, the 50th percentile concentration was added to the 50th percentile endogenous equivalent concentration and compared to the total equivalent concentration distribution for the non-smoking U.S. population. The findings demonstrate that impacts from nearby emission sources are small compared to mean background concentrations at nearby locations, and the total equivalent concentrations for exposed populations are generally indistinguishable from that of the 50th percentile for the non-smoking U.S. population.

## 1. Introduction

Measurable concentrations of ethylene oxide (EO) and its human metabolic precursor, ethylene, are ubiquitously present in the ambient air and are derived from industrial (e.g., stack and/or fugitive emissions) as well as natural (e.g., metabolism of certain plants and microbes and forest fires) and unregulated anthropogenic (e.g., tobacco smoke and fossil fuel combustion) sources [1,2,3,4,5,6]. As a result, virtually everyone is exogenously and endogenously exposed daily to some concentration of EO regardless of their occupation and geographic location [1,3,7,8,9,10]. Characterizing the potential health significance of environmental EO exposure for U.S. populations residing near emitting industrial facilities, where potential ambient concentrations may be above background levels, is challenging for two primary reasons. First, for the general population, ~94.5% of the mean total EO exposure (~2.9 parts per billion (ppb)) is from background endogenous ethylene metabolism rather than from exogenous inhalation of EO [3]. Second, there is a lack of practical utility of the current U.S. Environmental Protection Agency (EPA)’s inferred risk-specific concentrations (RSCs), which state that 0.0001 to 0.01 ppb is associated with an increase in inhalation cancer risk of 10^−6^ to 10^−4^, respectively [3,8,11]. Thus, this study evaluates alternative exposure metrics to address the immediate need for additional analytical tools to inform risk management decisions for populations exposed to industrial EO emissions.

The primary limitation of the U.S. EPA’s RSCs for EO is that they are so low relative to the amount and variability of general population exposures to EO that they have limited utility in distinguishing a health-significant increase in environmental exposure above background for near-facility populations. These RSCs are below current technology to measure EO in ambient air (current method detection limit (DL) for the U.S. EPA Method TO-15/TO-15A is ~0.02–0.09 ppb [12]); two orders of magnitude below EO ambient air concentrations not associated with industrial sources (mean of 0.13 ppb from recent U.S. EPA monitoring [1]); more than three orders of magnitude below airborne concentration equivalent to endogenously produced EO in the non-smoking U.S. population (mean concentration of 2.9 ppb [3]); and up to seven orders of magnitude below the exposure concentration (50,000–100,000 ppb [13]) for the 1930s–1970s sterilization operators and other highly exposed sterilization workers in the National Institute for Occupational Safety and Health (NIOSH) cohort that was used by the U.S. EPA in its EO cancer risk assessment.

Alternative exposure metrics to evaluate health-risk context associated with EO exposure were initially proposed in Kirman and Hayes [7] and Kirman et al. [3] (“endogenous equivalent concentration”) and subsequently by Sheehan et al. [8] (“total equivalent concentration”). Total equivalent concentration is the continuous EO exposure concentration extrapolated from hemoglobin adduct levels of 2-hydroxyethylvaline (HEV), a useful biomarker of total EO exposure regardless of exposure source and pathway. Endogenous equivalent concentration is the continuous exposure level specifically from the metabolically produced adducts (total minus exogenous fraction). HEV concentrations for the general U.S. population have been measured by the Center for Disease Control and Prevention (CDC) [9]. The conversion of HEV concentrations to equivalent continuous EO exposure concentrations in air is described in Kirman et al. [3]. An initial evaluation of the utility of these alternative equivalent exposure metrics for populations residing near emitting industrial facilities was presented in Sheehan et al. [8]. This initial evaluation was based on 50th and 90th percentile EO concentrations which were based on data collected from monitoring sites generally in proximity to emitting industrial facilities and frequently within the radius of adjacent residential areas.

To better inform risk management decisions, the present study focuses on using this total equivalent exposure metric to provide health-risk context for residential populations near selected emitting facilities in the state of Georgia, relying on publicly available data generated from both monitored and modeled EO concentrations in ambient air. It extends the analyses previously published [8] by including additional monitoring and modeled concentration data for three sterilization facilities previously evaluated, providing new modeled concentration data for five additional emitting industrial facilities, presenting national historical background EO concentration data and recent national background concentration data, and focusing the exposure analysis on the nearby residential populations to these emitting facilities. Specifically, this study includes the following components:

- Statistical characterization of ambient air EO concentrations at sites around emitting facilities in Georgia to assess whether they are above those at background sites;
- Comparison of mean monitored EO concentration at specific sites with mean background concentration to assess the potential contribution from facilities in Georgia;
- Comparison of monitored and modeled EO concentrations at closely located sites in Georgia to assess consistency between these two types of data;
- Comparison of U.S. national current and historical background EO concentrations to assess consistency in central tendency measures over time;
- Assessment of the relative health importance of excess EO concentrations for residential populations near emitting facilities in Georgia by comparing their estimated total EO exposure concentration to the distribution of that for the non-smoking U.S. population.

## 2. Materials and Methods

This section identifies the data and methods used to characterize endogenous equivalent and total equivalent concentrations and monitored and modeled EO concentrations in ambient air in the vicinity of emitting facilities along with representative regional and national background locations.

### 2.1. Equivalent EO Exposure Concentrations

CDC has published biomonitoring data for HEV concentrations in the blood of the general U.S. population collected over two sampling periods (2013–2014 and 2015–2016) for non-smokers (3841 persons) and smokers (93 persons) [9]. These data, which reflect a larger and more diverse population than assessed originally in Kirman and Hays [7], demonstrate that there are differences in HEV blood concentrations (and, therefore, total EO exposure), and depend on smoking status, age, and gender. These HEV concentrations (typically provided as picomole per gram hemoglobulin) can be converted to endogenous equivalent concentrations of EO based on the following equation [7]:

HEV (pmol/g Hb) = 10.9 × [EO, ppb continuous](1)

Endogenous equivalent concentrations reflect airborne concentrations of EO that are equivalent to the concentrations that are produced endogenously. The endogenous equivalent concentrations are calculated for EO based on: (1) HEV concentrations from CDC for non-smokers in the U.S. general population converted to continuous total exposure concentrations, and (2) background concentrations of HEV adjusted for contributions from the exogenous EO exposure pathway (via inhalation of ambient air). This process generates two exposure metrics: (1) total equivalent concentrations that represent continuous exposure concentrations from metabolic production of EO (i.e., endogenous equivalent concentration) plus inhaled EO in ambient air and (2) endogenous equivalent concentrations that represent continuous exposure concentrations produced metabolically from endogenous and exogenous ethylene (i.e., total equivalent concentration minus the mean exogenous exposure concentration). We previously reported distributions of these exposure metrics to describe the percentile concentrations of normal continuous exposure for the non-smoking U.S. population [8]. To refine the total concentration percentile distribution around the 50th percentile where most exposures are anticipated to occur, 40th and 60th percentile values were added to the original table (Table S1).

### 2.2. Available Data

#### 2.2.1. Monitored EO Concentrations in Ambient Air near Emitting Facilities and Associated Background Locations in Georgia

**Figure 1:**
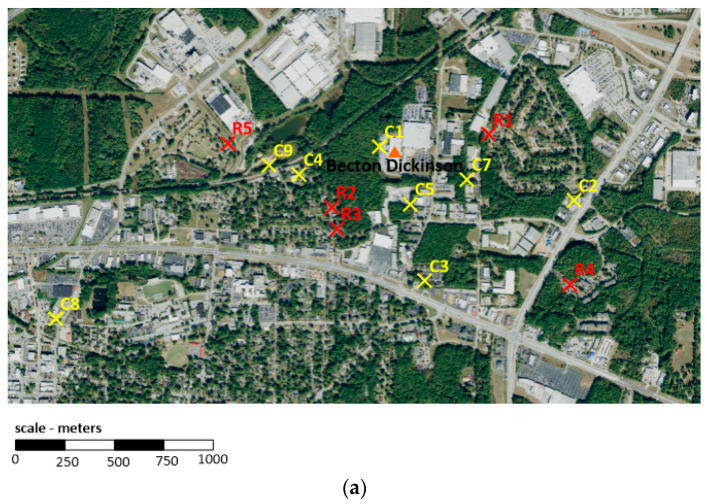

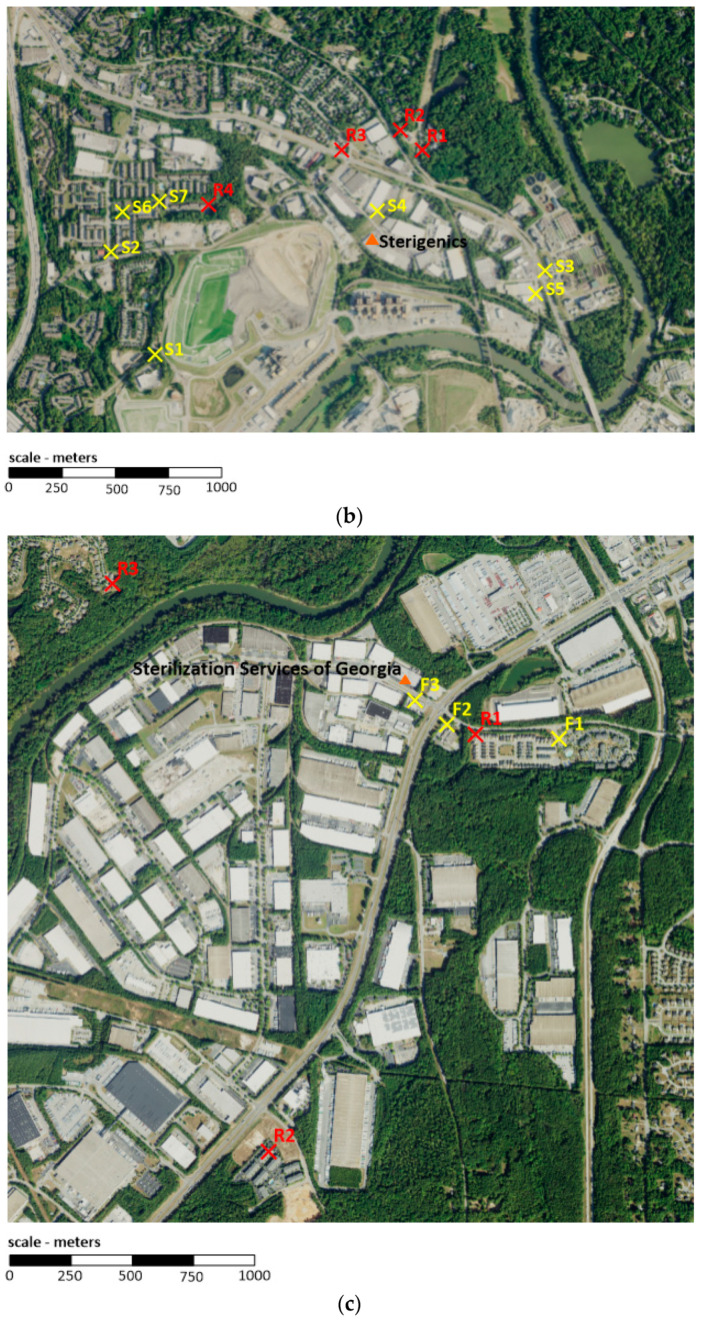
(**a**). Modeled (red) and monitored (yellow) sites around Becton Dickinson (Covington, Georgia). (**b**). Modeled (red) and monitored (yellow) sites around Sterigenics (Smyrna, Georgia). (**c**). Modeled (red) and monitored (yellow) sites around Sterilization Services (Atlanta, Georgia); sample site F4, which is not shown, is located more than ~5000 m northwest of Sterilization Services.

Under the direction of the Georgia Department of Natural Resources’ Environmental Protection Division (EPD) (Atlanta, USA), ambient air samples have been collected at selected locations in the vicinity of three sterilization facilities in Georgia (Becton Dickinson in Covington (as opposed to Madison, which is the location of another Becton Dickinson facility with corresponding modeling data only and later described in this paper), Sterigenics, and Sterilization Services) to characterize ambient EO concentrations in the vicinity of the facility [14,15,16,17,18] (see Table 1 and Figure 1a–c). EO concentrations for these samples were determined in accordance with the U.S. EPA methodology. All air samples were collected at multiple sampling sites within ~5500 m of these three facilities during multiple days per month and across all four seasons in 2019–2021 (except for Sterilization Services, which was 2020–2021). Background air samples were also collected in Georgia in both South Dekalb and General Coffee State Park. Given that these background air samples were collected at substantial distances from these facilities (>22,500 m) and in non-industrial settings, they are more likely representative of “state” or “regional” rather than “local” background EO concentrations. Six background air samples (<1% of the samples) with unusually high concentrations of EO (~1.5–5.4 ppb) were identified as potentially anomalous and removed by the authors prior to data analysis. Our prior published analysis [8] demonstrates that background EO concentrations in ambient air are generally no greater than ~1 ppb, which is the criterion that we used in the current paper for identifying outlying values. This is also generally consistent with the maximum concentration of EO in ambient air that has been reported by the U.S. EPA [10]. Approximately 3% of the samples were reported by the Georgia EPD as non-detect (ND). The DLs noted in the corresponding laboratory analytical reports were ~0.025–0.029 ppb.

#### 2.2.2. Modeled EO Concentrations in Ambient Air at Residential Locations near Emitting Facilities in Georgia

The Georgia EPD has prepared or compiled modeling evaluations for the three facilities described in Section 2.2.1 (see Table 2a and Figure 1a–c), as well as for five additional emitting facilities, including Becton Dickinson in Madison (sterilization), Kendall Patient Recovery (sterilization), Augusta University (university), Stepan (manufacturing), and ConMed (sterilized material warehouse), for which corresponding Georgia EPD monitoring data are not available (see Table 2b). These evaluations involved the running of the U.S. EPA AERMOD model [19] for 2014–2018 (except for Stepan, which was 2015–2019) of representative meteorological data. The models and methods used are consistent with regulatory applications where the goal is to identify the range of annual or short-term concentrations anticipated to occur in ambient air around a facility, though not necessarily the exact location or time of impacts. The modeled concentrations were identified for the closest receptors within multiple nearby residential neighborhoods around each facility. The closest residential receptor at each facility was generally at ~350–450 m. The most distant residential receptor modeled was at ~2000 m. Modeled concentrations generally decrease with distance but are not evenly distributed in all directions due to the frequency of prevailing winds relative to source and each site. Modeled concentrations were based solely on facility emissions and, thus, do not consider the contribution of background concentrations. We have reviewed the modeling memorandum but have not performed a detailed review of the modeling files or emissions inventories.

#### 2.2.3. Background EO Concentrations in Ambient Air in the United States

As summarized in Table 3, 24 h EO data are available through the U.S. EPA [10] for over 3700 ambient air samples that were recently (2018–2021) collected in 11 states across the U.S. as part of various monitoring programs. These data are provided on the U.S. EPA website as summary statistics. Two decades earlier (1999–2010), data were also available through the U.S. EPA Ambient Monitoring Archive for air samples collected in three U.S. states [10]; most of the samples were collected over 24 h, with a limited number of sites additionally reporting data for 3 h averaging times.

### 2.3. Data Analysis and Exposure Contextualization

The primary focus of this study are the Georgia EPD EO concentration monitoring and modeling data for sites around emitting facilities. Maps were generated to visualize sampling sites and their associated GA EPD-assigned descriptors (see Figure 1a–c). Prior to analysis of these data, we assigned monitoring data reported as ND with a value of DL divided by 2, consistent with our prior published analysis of these data and those collected in proximity to other sterilization facilities elsewhere in the U.S. [8] The mean, median, and standard deviation (SD) of the EO concentrations were calculated for each monitored site, after which a statistical comparison of background and non-background EO concentrations was performed. Given the skewed nature of these data, comparisons were performed using t-tests with log-transformed data and then, as a sensitivity analysis, Wilcoxon rank-sum tests of medians. To adjust for multiple comparisons and, therefore, minimize the potential for a statistically significant result due to chance, a Bonferroni correction was performed to the standard alpha of 0.05, resulting in a significance criterion of 0.0026 (i.e., 0.05 divided by 19 comparisons). Modeled concentrations were compared with monitored and background values to ensure they were reasonably consistent with observations, taking into consideration cases where monitored and background values were found to be not significantly different. Two exposure comparisons were performed for each of the eight Georgia facilities to provide context for interpreting the health implications of EO concentrations at nearby residential receptors: (1) comparison of the highest 5-year average modeled concentration to the mean and median background measured concentrations and (2) comparison of the estimated total residential population EO exposure (50th percentile endogenous + median exogenous background + highest annual average modeled concentration) with the distribution of the total equivalent EO concentrations for the non-smoking U.S. population.

A secondary objective of this study concerned the comparison of ambient air EO concentration data collected for current (2018–2021) and historical (1999–2010) monitoring programs. For the more recent data, which are available as summary statistics, NDs appear to have been assigned to a value of 0 ppb. Summary statistics for the earlier data are not provided by the original investigators. Contrary to the recent data, handling of the censored data in the earlier data set is not clear to us. Given this and differences in the DLs and frequency of reported NDs over time, there is some level of uncertainty in the alignment of the two datasets. As such, we decided that a formal statistical analysis was not appropriate and instead, consistent with the approach previously used by the Agency for Toxic Substances and Disease Registry (ATSDR) [1], range of medians by site and overall range formed the primary basis of the comparisons. Prior to analysis, two medians in the current monitoring data set were removed by the authors. One of these medians (associated with 13 samples) was removed because the median was markedly higher than the mean, which is unusual given the characteristic right-skewed distribution of EO air sample data as demonstrated here and previously [8]. The second median (associated with 4 samples) was removed because it had a value of 0 ppb, which is inconsistent with all other medians within this data set being greater than 0 ppb.

## 3. Results

### 3.1. Facility Vicinity and Background EO Concentrations in Georgia

#### 3.1.1. Monitoring Data

As shown in Table 4, measured ambient air EO concentrations are positively skewed for nearly all sample sites in Georgia. When the mean of the log-transformed concentration for each sample site within a facility data set was compared with the mean of the log-transformed concentration for the background sample sites in *t*-tests with the 0.05 significance adjusted by the number of comparisons (Bonferroni correction), significant differences were noted for four sites (C4, F1, F2 and F3). The findings were also equivalent when comparing medians using Wilcoxon rank-sum tests. Sample sites F1, F2, and F3 are in proximity to the same facility, Sterilization Services, and are among the highest measured EO concentrations, on average, relative to all other sample sites. The sites (and proximity to Sterilization Services) of F1 (~830 m), F2 (~340 m), and F3 (~110 m), which are generally in line with each other, southeast of the facility, were selected because, according to the Georgia EPD [16], they capture “primary downwind direction” of Sterilization Services. Notably, on average, EO concentrations that were measured at these three sites decrease with distance from Sterilization Services. Thus, the impact of Sterilization Services’ operations on ambient EO concentrations is characterized, to a large degree, by distance from the facility. The explanation for the finding at C4 is not entirely clear, especially because, according to the Georgia EPD, this sample site “Captures primary upwind and secondary downwind direction” of Becton Dickinson [15]. C4 is adjacent to and downwind of Georgia Subdivision, an active rail line currently used by 4.5 trains per day to transport freight between Atlanta and Augusta, Georgia [28]. Given that EO is an inadvertent byproduct of hydrocarbon fuel combustion, which is recognized as a potentially major background source of EO in ambient air [29,30], the operation of diesel engines associated with freight trains near C4 might be contributing to local background ambient concentrations, thereby confounding a clear interpretation of C4’s sample results. Taken together, these data indicate that only Sterilization Services’ operations are statistically significantly different to background EO concentrations at the identified monitoring sites downwind of this facility.

#### 3.1.2. Modeling Data

Table 5 presents a summary of the 5-year average modeled concentrations for each residential receptor site identified in the vicinity of the three sterilization facilities in Georgia with corresponding ambient EO monitoring data. These concentrations represent the modeled estimate of the facility EO contribution in absence of background. For each facility, emissions include controlled emissions along with fugitives emitted from building exhaust vents. Total annual emissions, as documented by the Georgia EPD, are also summarized. It should be noted that all the modeled 5-year average EO concentrations for residential sites are below the monitoring method DL, with the possible exception of the concentration for Sterilization Services site R1.

Table 6 presents a summary of the highest 5-year average modeled concentrations for the additional emitting facilities modeled by the Georgia EPD. Emissions sources for each facility are specific to the industry type and include both controlled and fugitive emissions. Total yearly emissions are also summarized.

#### 3.1.3. Comparison of Monitoring and Modeling Data

Modeled and monitored ambient air concentrations are subject to the influences of wind and other meteorological parameters that govern atmospheric stability and mixing. In general, concentrations for low level sources, such as those found at sterilization facilities, are expected to decrease with distance from the facility. These facilities all have low level sources that are generally similar in character. As a result, impacts at specific distances from a facility are often linearly related to emission rates. The frequency of hourly impacts will not be consistent for all wind directions due to the prevailing wind patterns at a given site, and 5-year average concentrations may as a result show directional variability. Based on the methodology used, model results are expected to be representative of concentrations for the residential regions around each facility.

Monitoring and modeling sites near the Becton Dickinson, Sterigenics, and Sterilization Services facilities are shown in Figure 1a–c, respectively. These figures show that none of the monitoring and modeling sites were co-located; however, some qualitative comparisons can be made. Since most of the monitored concentrations around the considered sterilization facilities are not significantly different from regional background levels, it is expected that the modeled concentrations at sites near these monitors would be small. This is proven by the modeling data available through the Georgia EPD. At the Becton Dickinson facility (Figure 1a), monitor site C7 recorded a mean value of 0.28 ppb, which is determined to not be significantly different from the background concentration of 0.18 ppb. The modeling analysis at residential receptor R1, which is located at a similar distance in the eastern sector, shows a comparatively small contribution of ~0.01 ppb from the facility. A similar situation is present for Sterigenics (Figure 1b) where monitor S7 lies in the same direction, but slightly closer to the facility than modeled receptor R4 and shows a mean concentration of 0.22 ppb. Again, the modeling predicts a comparatively small contribution from the facility of 0.005 ppb. Sterilization Services has three nearby monitor sites with concentrations that were determined to be significantly above background (Figure 1c). These monitors (F3, F2, and F1) lie at 110, 340, and 830 m, respectively, from the facility and show respective concentrations of 1.5 ppb, 0.56 ppb, and 0.29 ppb. Modeled receptor R1 lies between F1 and F2 and is shown to contribute 0.037 ppb due to the facility-specific emissions. This is a larger value than seen at the other modeled facilities, consistent with monitored concentrations being measurably above background.

**Figure 2:**
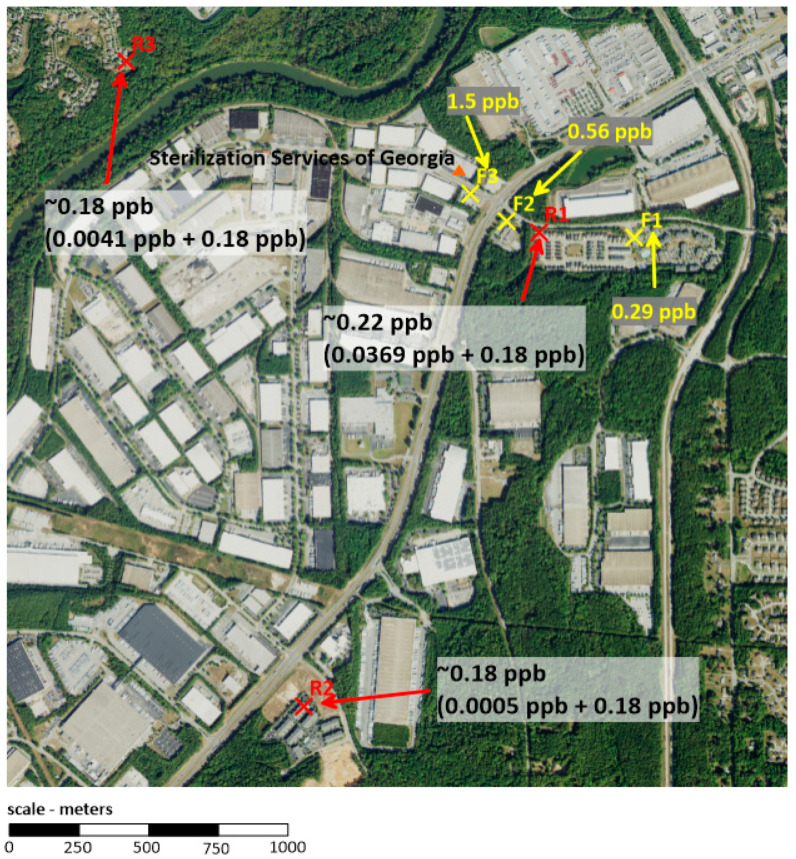
Adjusted EO concentrations at modeled sites (red: modeled + mean background, 0.18 ppb) and measured EO concentrations at monitored sites (yellow) around Sterilization Services (Atlanta, Georgia).

A specific comparison of Sterilization Services EO concentration data is depicted in Figure 2. EO concentrations diminish rapidly (~3-fold) in the relatively short distance between locations F3 and F2. The modeled and monitored EO concentrations at R1 and F1, respectively, are similar in value and are approximately half the concentration at F2 located closer to the facility, indicating that beyond some distance from the source there is a marginal contribution of facility emissions to background concentrations. Additionally, the modeled EO concentration at R1 adds measurably to mean background EO concentration while modeled EO concentrations at distant sites R2 and R3 do not, as would be expected.

### 3.2. Current and Historical Background EO Concentrations in the U.S.

As shown in Table 7, the range of the U.S. EPA-reported medians for the current (2018–2021) measurements was 0.03–0.33 ppb. This finding prompted a further investigation by the authors, given the range of means reported for an earlier version (2018–2019) of these measurements was 0.08–0.22 ppb [1,8]. For the current reported measurements, the upper-bound of the range of means was 0.34 ppb (data not reported in Table 7). While a discrepancy in the upper-bound of the range of means over time might be attributed, in part, to the expansion of the data set, a review of the current measurements revealed there is potentially more at issue. Notably, ~24% of the medians are higher than their respective means, which is somewhat unusual given that EO air sample concentration data are typically characterized by a right-skewed distribution. Thus, there appears to be some level of uncertainty associated with these reported summary statistics, limiting the authors’ ability to directly compare them with the historical (1999–2010) EO measurements. Nevertheless, if we accept the reported summary statistics for the current measurements as true, it is noteworthy that ~80% of all medians were 0.15 ppb or less, which is consistent with the range of medians for the historical measurements. Taken together, there generally appears to be an overlap in median background EO concentrations between these two periods.

### 3.3. Risk Management Context for Near Facility Potential Population Exposure Concentrations

**Figure 3:**
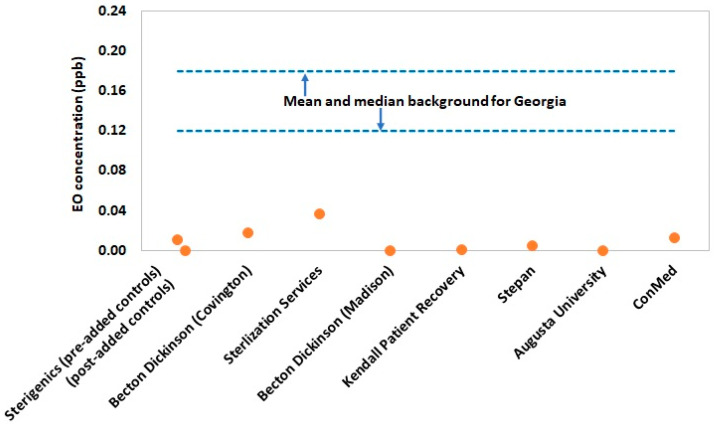
Highest modeled 5-year average EO concentration for all residential receptors by facility (in absence of background contribution) relative to the mean background EO concentration for Georgia (0.18 ppb). One facility (Sterigenics) had modeling data corresponding to two different time periods, which were delineated by the addition of supplemental EO emission controls.

**Figure 4:**
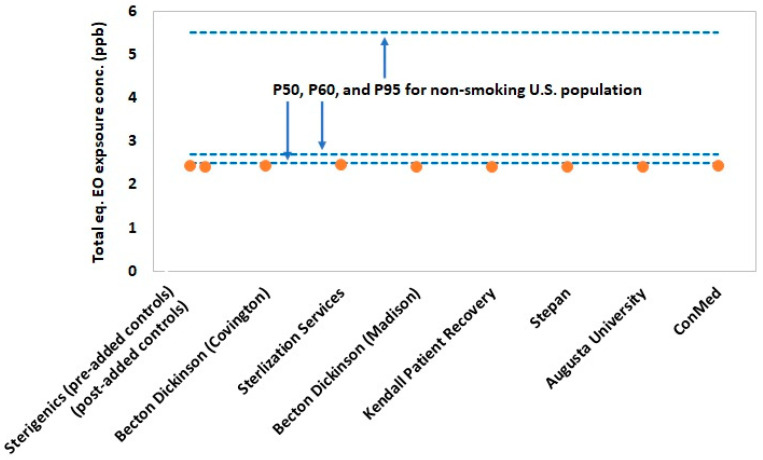
Estimated total equivalent exposure for the highest 5-year average modeled EO concentration for all residential receptors by facility ((50th percentile endogenous equivalent for the non-smoking U.S. population, or 2.3 ppb) + (50th percentile background EO concentration for Georgia, or 0.12 ppb) + (highest 5-year average modeled EO concentration for all residential receptors by facility)) relative to that of the 50th, 60th, and 95th percentiles of the non-smoking U.S. population (2.5, 2.7, and 5.5 ppb, respectively).

Figure 3 and Figure 4 summarize the values for exposure metrics that provide context for interpreting the health significance of potential EO exposure concentrations for residential populations in the vicinity of the eight emitting facilities evaluated in the present study. In nearly all cases, the highest modeled annual ground level concentration at residential sites in the vicinity of the emitting facilities were less than 10% of the mean background EO concentration for Georgia (Figure 3). Thus, the emitting facilities are contributing minimally above state background levels and, therefore, would not contribute a significant health risk. The lack of health significance of these small, estimated facility contributions to background EO is reinforced by the lack of statistically significant increases in EO concentration for monitored sites close to two of the three sterilization facilities (Sterigenics and Becton Dickinson). More importantly, the total exposure metric that evaluates the significance of total exposure by adding the highest residential site 50th percentile EO concentration to the median background concentration and the non-smoking U.S. population 50th percentile endogenous concentration, showed that all environmental EO concentrations at nearby residential sites are negligible contributors to population total exposure (Figure 4). In fact, residential total exposure concentrations are virtually indistinguishable from the 50th percentile non-smoking U.S. population total equivalent EO concentration. This analysis indicates that there is a substantial margin of exposure (~2.5 ppb) between modeled total equivalent concentrations for these and the upper-bound for the normal total exposure distribution of the non-smoking U.S. population.

Based on the EO monitoring data, only the three sites (F1, F2, and F3) downwind of Sterilization Services operation had average EO concentrations significantly above background concentrations. The total exposure concentrations at these monitoring sites near this facility are 2.52 ppb (~50th percentile of the non-smoking U.S. population) for F1, 2.75 ppb (~60th percentile of the non-smoking U.S. population) for F2, and 3.28 ppb (~75th percentile of the non-smoking U.S. population) for F3. One additional site (C4) near Becton Dickinson was significantly above background on average, but as noted previously, this finding is possibly not driven by facility emissions. The total exposure at this monitoring site near this facility is 2.46 ppb (~50th percentile of the non-smoking U.S. population). The collective total exposure concentrations for these four monitoring sites are well within that of the total equivalent concentrations for the U.S. non-smoking population.

## 4. Discussion

EO is a unique chemical in the context of general population risk assessment because one’s total EO exposure is driven almost entirely by endogenous metabolism [3], with the balance attributed to comparatively smaller exogenous exposures via inhalation from industrial, natural, and/or unregulated anthropogenic sources of EO [1,2,3,4,5,6]. We are aware of no other industrial chemical that exhibits a comparable exposure pattern by source in the general population. To date, regulatory criteria based on the U.S. EPA’s RSCs [11] are used to benchmark ambient concentrations of EO and, in turn, inform risk management decisions concerning emitting industrial sources. However, the U.S. EPA’s RSCs are not a practical risk management tool, namely because they are below current measurement technology and below concentrations measured in ambient air at background locations and substantially below equivalent airborne concentrations from human metabolism [1,3,12]. Consequently, characterizing the potential health significance of facility emissions-related increases in ambient EO concentrations is particularly challenging and unprecedented from a regulatory perspective. Pragmatic science-based exposure metrics, such as total equivalent concentration [3,8] based on total exposure and able to account for additional industrial facility-related emissions contributions, are needed to fill the current risk assessment void. The application of this exposure metric is consistent with the interpretation of clinical metrics for which disease risk is not considered to be significantly increased until the values are above the range for the healthy population defined by individual variability within the population.

The results of the present study demonstrate that total exposure to EO for residential populations situated near these emitting facilities are indistinguishable from that of the 50th percentile of the U.S. general non-smoking population. This finding is reinforced by the statistical analysis of monitored EO concentrations for two of the three sterilization facilities, many of which are from sites closer to the emitting facility than represented for residents in modeling. This analysis showed that mean facility monitoring site concentrations were indistinguishable from mean background concentrations with one exception, where an alternate source was viably identified. The total exposure concentration at monitoring sites near Sterilization Services with measurable facility contributions also are well within the normal total equivalent concentration range of the non-smoking U.S. population and consistent with our previous study [8]. A substantial margin of additional facility-related exposure (~2.5 ppb or nearly 100-fold increase in concentration at the residential site with the highest 5-year average annual concentration) would be needed to exceed the 95th percentile of the distribution of the non-smoking U.S. population. It, therefore, seems unlikely that current or recent past EO concentrations at the residential sites evaluated in the present study have significantly exposed residents from a health-risk perspective.

The finding of general consistency when comparing long-term EO concentrations at monitoring sites with annualized concentrations at geographically close modeling sites is reassuring as this supports the potential utility of modeling as a more cost-effective alternative to monitoring in generalizing concentration estimates considering longer-term meteorological conditions and providing historical concentration estimates when no monitoring data are available.

Furthermore, given that there will likely be an interest in estimating historical EO concentrations at specific sites around emitting facilities, it appears that there is overlap in median historical and current national background EO concentrations, despite differences in the reported DLs and handling of data below the DLs over time, and potential uncertainty associated with the reported summary statistics for the current measurements.

We relied upon publicly available data sets that were generated by state and federal agencies (CDC, Georgia EPD, and the U.S. EPA) (Atlanta and Washington, DC, USA, respectively) and did not perform an independent evaluation to confirm their reliability, but we have confidence in these data sets given that well-accepted methodologies were employed by the original investigators. Our study was restricted by the sample sites selected by the original investigators, which may have limited the statistical power of our comparisons due to site-specific sample size and variability of measured EO concentrations. Our study was also restricted by the geographic location of modeling receptor sites, which were not collocated with sampling sites and, in turn, somewhat limited our confidence to make a direct comparison of the monitoring and modeling data. Nevertheless, the data set for Georgia is the largest and most robust of all U.S. states for which ambient air EO near emitting facilities has been evaluated [8]. Collectively, our present findings for Georgia and our prior published findings for other U.S. states [8] suggest a pattern of negligible contribution of EO from facility emissions to the total exposure of near residential populations. Additional monitoring and/or modeling of other emitting facilities will be helpful in confirming this observed pattern.

Overall, considering current risk assessment limitations for risk management, the findings of this analysis indicate that the total equivalent concentration, importantly based on a systemic dose metric for EO exposure, is perhaps the most useful currently available tool for contextualizing the health significance of EO exposures for populations residing near emitting facilities.

## 5. Conclusions

Comparing total EO exposure concentrations to the distribution of total equivalent concentration metrics for the U.S. non-smoking population provides a useful evaluation method for assessing the health significance of EO exposure for populations residing near emitting industrial facilities and informing risk management decisions, particularly with the current risk assessment limitations. The present study shows that EO emissions for the Georgia facilities evaluated contribute negligibly to total EO exposure of nearby residential populations. Our previous and current evaluations show that excess ambient concentrations above background from facility EO emissions are relatively low and do not warrant risk management actions to protect nearby populations.

## Figures and Tables

**Table 1:** Summary of publicly available datasets concerning measurement of ambient air EO concentrations in Georgia in the vicinity of three sterilization facilities and at background locations.

Facility	Location	*n* (Sites) ^1^	Dist. ^2^
Sterigenics [14]	Smyrna	400 (7)	~260–1640
Becton Dickinson [15]	Covington	441 (8)	~110–2370
Sterilization Services [16]	Atlanta	190 (4)	~110–5630
Background [17]	General Coffee	43 (1)	>290,000
Background [18]	South DeKalb	144 (2)	>22,500

^1^ Total number of samples (total number of sampling sites); ^2^ estimated distance of sample sites from facility in meters.

**Table 2:** (**a**). Summary of publicly available datasets concerning modeling of facility-specific ambient air EO concentrations in Georgia in the vicinity of three sterilization facilities. (**b**). Summary of publicly available datasets concerning modeling of facility-specific ambient air EO concentrations in Georgia in the vicinity of additional facilities.

(**a**)			
**Facility**	**Location**	**Residential Sites ^1^**	**Dist. ^2^**
Sterigenics [20]	Smyrna	4	~440–790
Becton Dickinson [21]	Covington	5	~430–1100
Sterilization Services [22]	Atlanta	3	~360–2030
(**b**)			
**Facility**	**Location**	**Residential Sites ^1^**	**Min. Dist. ^2^**
Becton Dickinson [23] ^3^	Madison	6	~1760
Kendall Patient Recovery [24] ^3^	Richmond	3	~1060
Stepan [25] ^4^	Barrow	1	~550
Augusta University [26] ^5^	Richmond	1	~250
ConMed [27] ^6^	Douglas	1	~450

(**a**) ^1^ Total number of residential sites reported; ^2^ estimated distance of receptor in meters. (**b**) ^1,2^ As with Table 2a; ^3^ sterilization facility; ^4^ manufacturing facility; ^5^ university; ^6^ warehouse facility.

**Table 3:** Summary of publicly available datasets concerning measurement of background ambient air EO concentrations associated with various current and historical monitoring programs in the U.S.

States ^1^	Years	*n* (Sites) ^2^
AZ, CO, FL, IL, KY, MI, MO, NJ, NY, UT, WA	2018–2021 ^3^	3706 (36)
MA, NH, RI	1999–2010	5726 (21)

^1^ AZ, Arizona; CO, Colorado; FL, Florida; IL, Illinois; KY, Kentucky; MA, Massachusetts; MI, Michigan; MO, Missouri; NH, New Hampshire; NJ, New Jersey; NY, New York; RI, Rhode Island; UT, Utah; WA, Washington; MA ^2^ total number of samples (total number of sampling sites); ^3^ a limited subset of sites also had data available during 2011–2016, but due to small sample size were not included.

**Table 4:** Measures of central tendency and spread for ambient air EO concentrations in Georgia in the vicinity of three sterilization facilities and at background locations.

Facility	Sample Site (*n*) ^1^	Distance ^2^	Mean (SD) ^3^	P50
			ppb	ppb
Sterigenics	S1 (91)	~1420	0.24 (0.25)	0.15
	S2 (88)	~1640	0.24 (0.40)	0.14
	S3 (94)	~910	0.25 (0.26)	0.16
	S4 (101)	~260	0.24 (0.18)	0.19
	S5 (3)	~880	0.55 (0.51)	0.33
	S6 (7)	~1570	0.15 (0.16)	0.13
	S7 (16)	~1350	0.22 (0.22)	0.15
Becton Dickinson (Covington)	C1 (10)	~110	0.40 (0.45)	0.21
	C2 (98)	~1020	0.31 (0.81)	0.18
	C3 (101)	~730	0.21 (0.17)	0.16
	C4 (109) ^4^	~800	0.27 (0.23)	0.16
	C5 (94)	~330	0.21 (0.16)	0.18
	C7 (18)	~400	0.28 (0.26)	0.20
	C8 (6)	~2370	0.09 (0.04)	0.09
	C9 (5)	~910	0.13 (0.08)	0.13
Sterilization Services	F1 (75) ^4^	~830	0.29 (0.25)	0.22
	F2 (83) ^4^	~340	0.56 (0.49)	0.45
	F3 (13) ^4^	~110	1.5 (1.09)	0.98
	F4 (19)	~5360	0.11 (0.10)	0.06
Background	Overall (187)	>22,500	0.18 (0.16)	0.12

^1^ Sample sites labeled by the Georgia EPD and total number of samples collected at that sample site; ^2^ estimated distance of sample sites from facility in meters; ^3^ SD, standard deviation; ^4^ significantly different from background when data were first log-transformed and then compared in t-tests, incorporating a Bonferroni correction (alpha = 0.0026) to adjust for multiple comparison. Similar results were observed when instead comparing medians in Wilcoxon rank-sum tests, again with a Bonferroni correction.

**Table 5:** Modeled residential ambient air EO concentrations for the three sterilization facilities in Georgia with corresponding with ambient EO monitoring data.

Facility	Emissions	Receptor Site ^1^	Distance ^2^	5-Year Average ^3^
	lbs/Year			ppb
Sterigenics	206.0	R1	~470	0.0111
		R2	~520	0.0083
		R3	~450	0.0094
		R4	~790	0.0050
Becton Dickinson (Covington)	657.4	R1	~470	0.0156
		R2	~430	0.0050
		R3	~500	0.0033
		R4	~1100	0.0056
		R5	~860	0.0067
Sterilization Services	1339.5	R1	~370	0.0369
		R2	~2030	0.0005
		R3	~1260	0.0041

^1^ Receptor sites labeled by the Georgia EPD; ^2^ estimated distances of receptor from facility in meters; ^3^ 5-year average concentrations based on modeling do not include contribution from background.

**Table 6:** Modeled residential ambient air EO concentrations for the additional sterilization, manufacturing, and warehouse facilities in Georgia without corresponding ambient EO monitoring data.

Facility	Emissions	Minimum Distance ^1^	5-Year Average ^2^
	lbs/Year		ppb
Becton Dickinson (Madison)	49.8	~1760	0.0001
Kendall Patient Recovery	199.7	~1060	0.0009
Stepan	82.6	~550	0.0052
August University	0.16	~250	0.0004
ConMed	466.4	~350	0.0133

^1^ Estimate distance of receptors from facility in meters; ^2^ 5-year average concentrations based on modeling do not include contribution from background.

**Table 7:** Range of medians and overall range of background ambient air EO concentrations associated with various current and historical monitoring programs in the U.S.

Description	Year	Range of Medians	Overall Range
		ppb	ppb
Current (11 states) Historical (3 states)	2018–2021	0.03–0.33	0–0.91 ^1^
	1999–2010	0.07–0.15	0–2.95 ^1^

^1^ Low-end is 0 ppb because the U.S. EPA appears to have assigned NDs a value of 0 ppb.

## Data Availability

Publicly available datasets were analyzed in this study. These data can be found in the References section.

## Supplementary Materials

The following supporting information can be downloaded at: https://www.mdpi.com/article/10.3390/ijerph19063364/s1, Table S1: Total equivalent and endogenous equivalent EO air concentrations (ppb) using HEV levels (pmol/g Hb) in percentiles of nonsmoker population in the United States, as originally reported by Kirman et al. [3], based on CDC [9] data.
