# Supplementary material for: Monitored and Modeled Ambient Air Concentrations of Ethylene Oxide: Contextualizing Health Risk for Potentially Exposed Populations in Georgia
Source: Int J Environ Res Public Health. 2022 Mar 12;19(6):3364. doi: 10.3390/ijerph19063364 (PMC8954488; doi:10.3390/ijerph19063364)
Supplement: Supplementary file 1 [file ijerph-19-03364-s001.zip › ijerph-1596645-supplementary.pdf]

Table S1. Total equivalent and endogenous equivalent EO air concentrations (ppb) using HEV levels (pmol/g Hb) in percentiles of nonsmoker population in the United States, as originally reported by Kirman et al. [3], based on CDC [9] data.

Percentile	HEV_{unadj} [1]	Total Eq. [2]	HEV_{adj} [3]	Endog. Eq. [4]
	pmol/g Hb	ppb	pmol/g Hb	ppb
P5	13.4	1.3	11.2	1.0
P10	16.0	1.5	13.8	1.3
P25	20.8	1.9	18.6	1.7
P40	23.8	2.2	21.6	2.0
P50	27.0	2.5	24.8	2.3
P60	29.0	2.7	26.8	2.5
P75	35.1	3.2	32.9	3.0
P90	47.5	4.4	45.3	4.2
P95	60.1	5.5	57.9	5.3

[1] HEV_{unadj}, HEV level from exogenous + endogenous sources; [2] total equivalent EO concentration from endogenous and background exogenous source; [3] HEV_{adj}, HEV level from endogenous sources only; and [4] endogenous equivalent EO concentration from individual metabolism.
